# Anatomical variation analysis of left upper pulmonary blood vessels and bronchi based on three-dimensional reconstruction of chest CT

**DOI:** 10.3389/fonc.2022.1028467

**Published:** 2022-11-21

**Authors:** Youjun Deng, Songhua Cai, Chujian Huang, Wenyi Liu, Longde Du, Chunguang Wang, Ran Jia, Shengcheng Lin, Xin Yu, Xiangyang Yu, Yikun Yang, Chenglin Yang, Hongbo Zhao, Zhe Wang, Lixu Wang, Kai Ma, Zhentao Yu, Xiaotong Guo

**Affiliations:** National Cancer Center, National Clinical Research Center for Cancer, Cancer Hospital & Shenzhen Hospital, Chinese Academy of Medical Sciences and Peking Union Medical College, Shenzhen, China

**Keywords:** anatomical variation, CT, NSCLC, pulmonary, segmentectomy, three-dimensional, VATS

## Abstract

**Background:**

With its growing popularity and potential outcome, preoperative three-dimensional reconstruction of chest computed tomography (CT) has been widely used in video-assisted thoracic surgery (VATS) segmentectomy for treating non-small cell lung cancer (NSCLC). This study aimed to summarize the experience of anatomical variation analysis of left upper pulmonary blood vessels and bronchi based on the three-dimensional reconstruction of chest CT.

**Materials and methods:**

A total of 103 patients with early-stage NSCLC were chosen to undergo VATS segmentectomy based on preoperative three-dimensional reconstruction of chest CT in our institute from September 2019 to July 2022. Data such as clinical characteristics and variations in blood vessels and bronchi were reviewed in this study.

**Results:**

The branches of the left lingular pulmonary artery may mutate into the LS1 + 2 + 3. A1 + 2 has four subtypes. The distribution of variation is relatively balanced, and the most common variation is type I (35/103, 33.9%). Most lingular arteries originate from the oblique cleft side of the lingular bronchus (79/103,76.7%). Most V(1 + 2)c* are small developments (70/103, 68.0%). The venous return of the proper segment mainly depends on V(1 + 2)b + c. The variation in the left upper lobe bronchus is complex. The most common variant is the bifurcation type (type A to G, 92/103, 89.3%) and bifurcation type A (62/103, 60.2%). The posterior apical segment artery of the left upper lobe is not accompanied by its bronchus.

**Conclusions:**

The variation types of blood vessels and bronchus in the upper lobe of the left lung are complex. Preoperative CT-based three-dimensional reconstruction of pulmonary arteries, veins, and bronchi is of great significance. It can help understand the variations, accurately locate lesions before the surgery, and effectively plan surgeries.

## Introduction

Video-assisted thoracic surgery (VATS) lobectomy has become the standard procedure for treating early-stage non-small cell lung cancer (NSCLC) (1). Currently, for some specific early-stage lung cancer, VATS segmentectomy through fine anatomy can not only achieve the same therapeutic effect as VATS lobectomy but also further preserve normal lung tissue, thereby maximizing the preservation of lung function (2). However, the surgical difficulty and complexity of VATS segmentectomy are much higher than those of VATS lobectomy. Variations in the blood vessels and bronchi within the lung also pose new challenges for VATS segmentectomy.

Left upper lobe lung cancer is common in clinical practice. The anatomical variations in the left upper lobe are relatively common and complex, which greatly increases the difficulty in VATS segmentectomy of the left upper lobe. Knowledge of left upper vascular and bronchial anatomical variations plays an important role in the planning and implementation of left upper lobe VATS segmentectomy. Therefore, our center performed a three-dimensional reconstruction of thoracic CT data of 103 patients with left upper pulmonary nodules. The anatomical variations in the left upper pulmonary artery, vein, and bronchus in 103 patients were summarized in this study. The findings might provide a clinical reference for VATS left upper segmentectomy.

## Methods

From September 2019 to July 2022, 103 patients were enrolled from the Department of Thoracic Surgery, National Cancer Center/National Clinical Research Center for Cancer/Cancer Hospital & Shenzhen Hospital, Chinese Academy of Medical Sciences, and Peking Union Medical College, Shenzhen, China. The age ranged from 28 to 78 years, with a median age of 55 years. Of these patients, 39 were male and 64 were female. The diameter of the lesions in the left upper lobe ranged from 2.3 mm to 46.9 mm, with a median diameter of 7.9 mm. All patients underwent a chest CT plain scan (GE revolution CT). After collecting CT data, Mimic 24.0 software was used for three-dimensional reconstruction. Variations in the left upper pulmonary artery, veins, and bronchi were analyzed and summarized.The inclusion and exclusion criteria of this study:

### Inclusion criteria

1.Main target lesion, located in left upper lung, ≥6mm at least;

2.Patients were candidates for segmental resection:①.Poor pulmonary reserve or other major comorbidity that contraindicates lobectomy.② pure ground glass opacity or Nodule has ≥50% ground-glass appearance on CT;Radiologic surveillance at least ≥6 months;Frozen pathology confirmed AIS or MIA

3.Non small cell lung cancer confirmed by paraffin pathology

Exclusion criteria:

1.Preoperative three-dimensional reconstruction indicates that the lesion is located in the central area of the lung lobe (e.g., the lesion is adjacent to the bronchial opening of the lung lobe), operator thinks it is not suitable to perform segmental resection

2.Patient refused to accept segmentectomy

3.History of left lung surgery

4. History of pulmonary tuberculosis

5.Combined with other malignant tumors

## Results

### Variations in the left upper pulmonary artery

The variations in the left upper pulmonary artery are listed in **Table 1**.

**Table 1:** Variations in the left upper pulmonary artery (L, left; S, segment; A, artery).

	Type	Subtype	Patients
Variations in the left upper pulmonary artery	The branches of the lingular artery entered the LS1 + 2 and LS3	The lingular artery branches entered the LS3	13
		The lingular artery branches entered LS1 + 2	7
		The lingular artery entered both LS1 + 2 and LS3	1
	The artery co-trunk of LS1 + 2	Type I: A(1 + 2)a, A(1 + 2)b, and A(1 + 2)c were separate branches	35
		Type II: A(1 + 2)a and A(1 + 2)b were co-trunks, and A(1 + 2)c was a single branch	33
		Type III: A(1 + 2)a was a single branch, and A(1 + 2)b and A(1 + 2)c were co-trunks	21
		Type IV: A(1 + 2)a, A(1 + 2)b, and A(1 + 2)c were co-trunks	14
	The occurrence and walking of the lingular artery (A4 + A5)	A4 originated from the mediastinal side of the lingular bronchus, while A5 originated from the oblique cleft side of the lingular bronchus	17
		A4 + 5 originated from the mediastinal side of the lingular bronchus	7
		A4 + 5 originated from the oblique cleft side of the lingular bronchus	79

**Figure 1:**
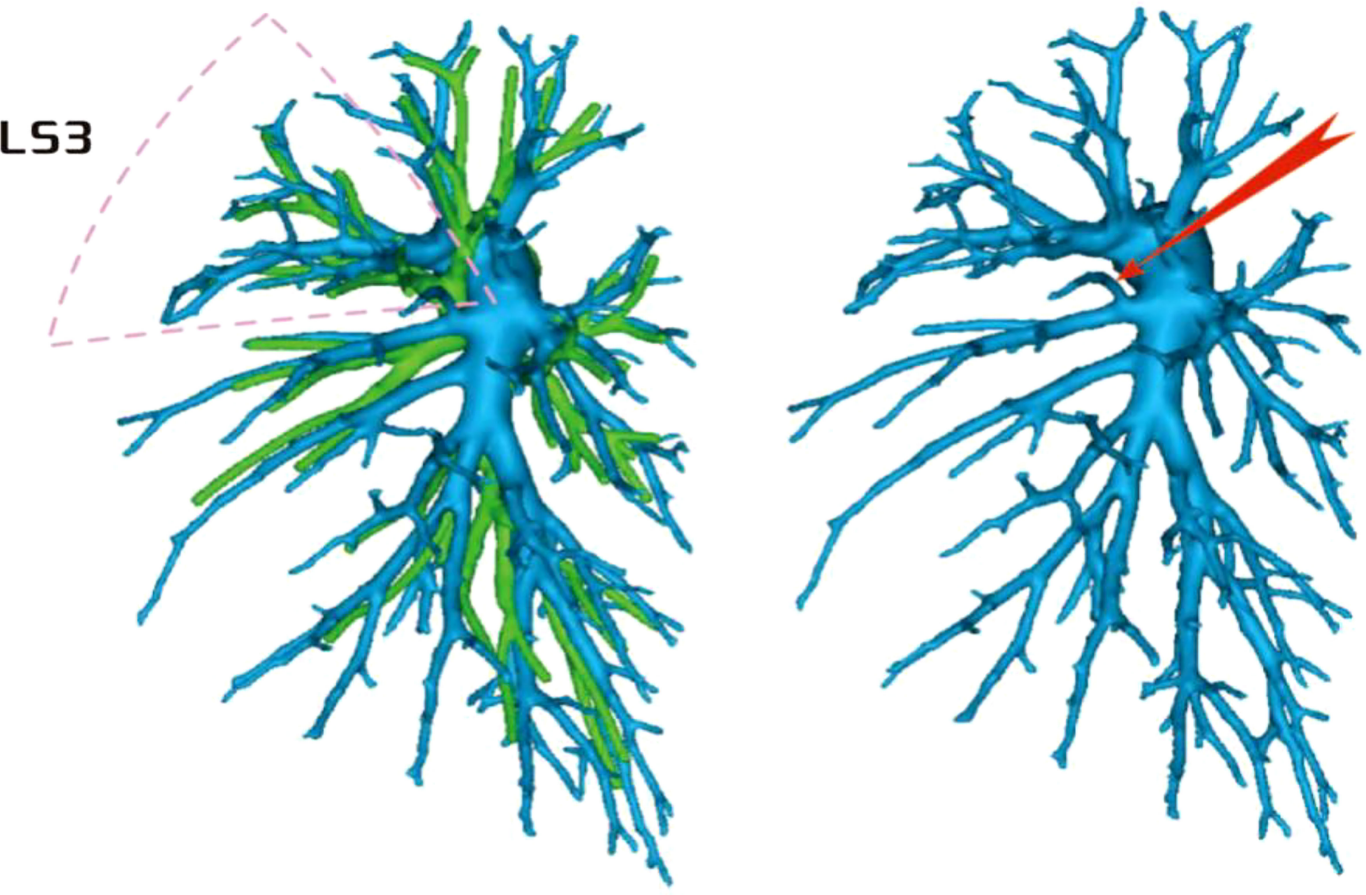
Dotted line is the LS3 area, and the red arrow indicates the branch of the lingular artery into LS3.

**Figure 2:**
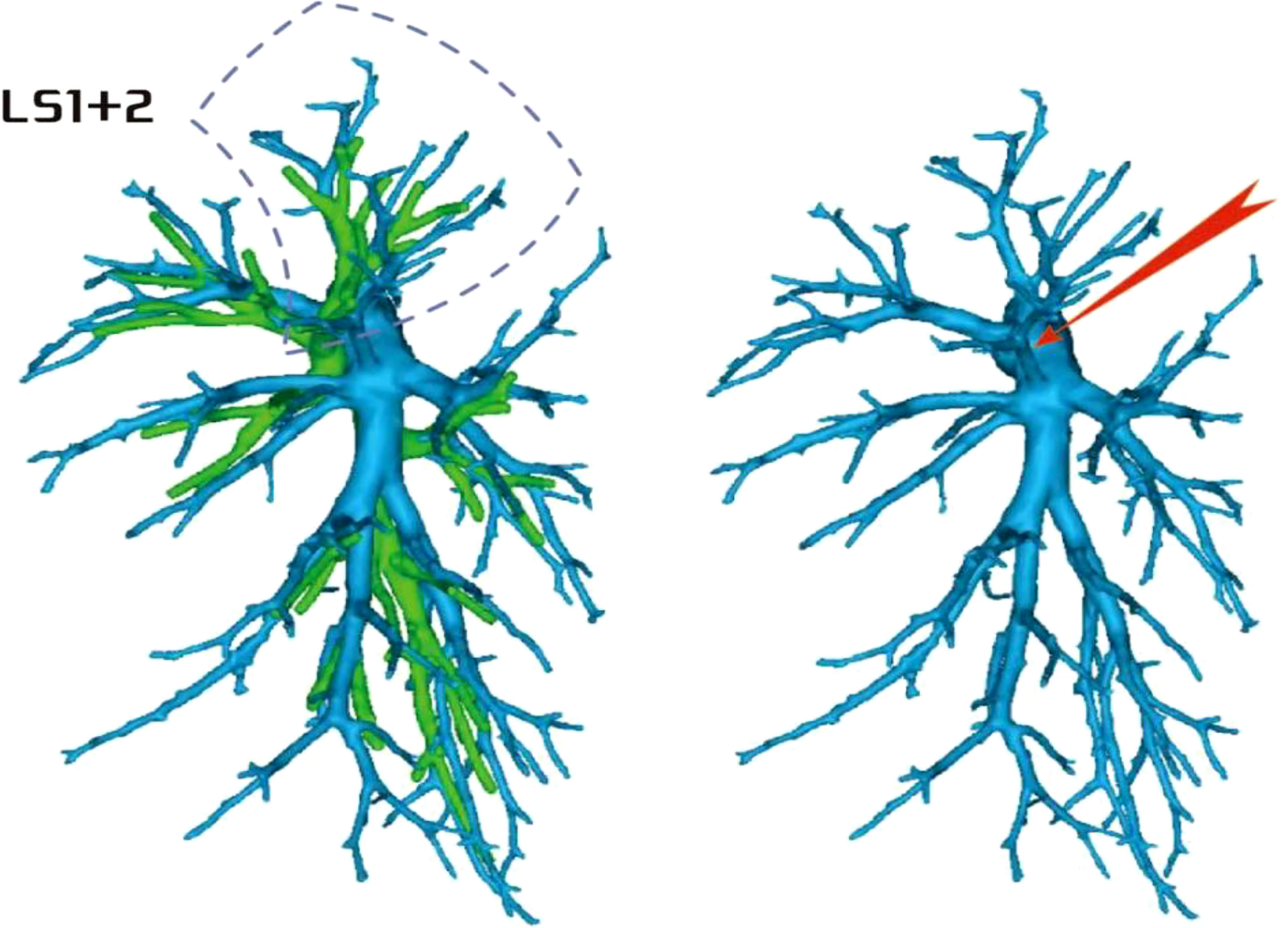
Dotted line is the LS1 + 2 area, and the red arrow indicates the branch of the lingular artery into LS1 + 2.

**Figure 3:**
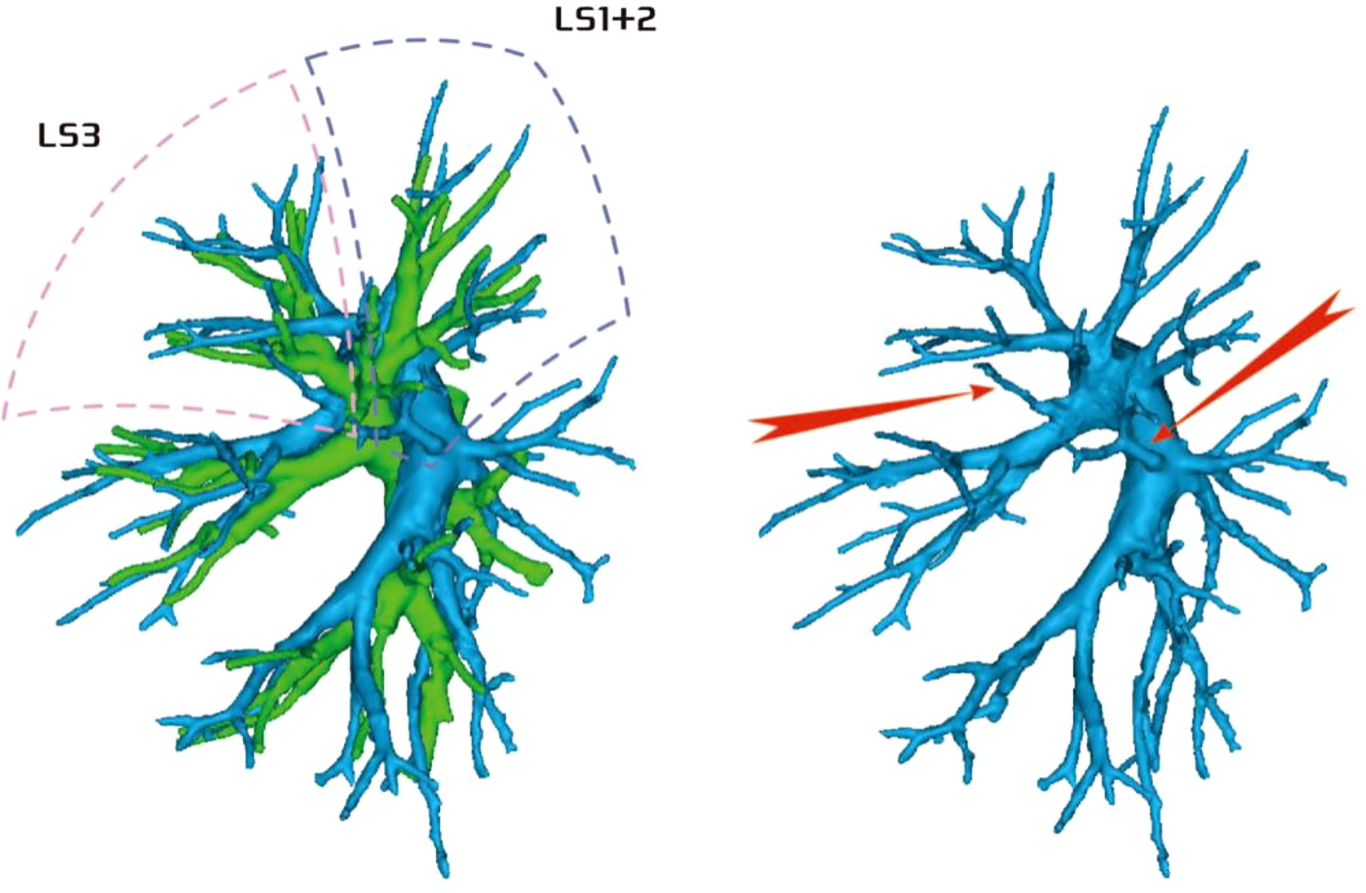
Dotted line is the area of LS3 and LS1 + 2. The red arrow indicates the branch of the lingular artery entering LS1 + 2 and LS3 at the same time.

Among the 103 patients, the total incidence rate of the branches of the left upper lingular artery entering the LS1 + 2 and LS3 segments (21/103, 20.3%) had the following three variations: (1) The lingular artery branches entered the LS3 area in 13 patients (13/103, 12.6%) (representative cases are shown in **Figure 1**). (2) Seven patients have lingular artery branches entering the LS1 + 2 area (7/103, 6.8%) (representative cases are shown in **Figure 2**). (3) In one patient, the branches of the lingular artery entered both LS1 + 2 and LS3 areas (1/103, 0.9%) (case is shown in **Figure 3**).

**Figure 4:**
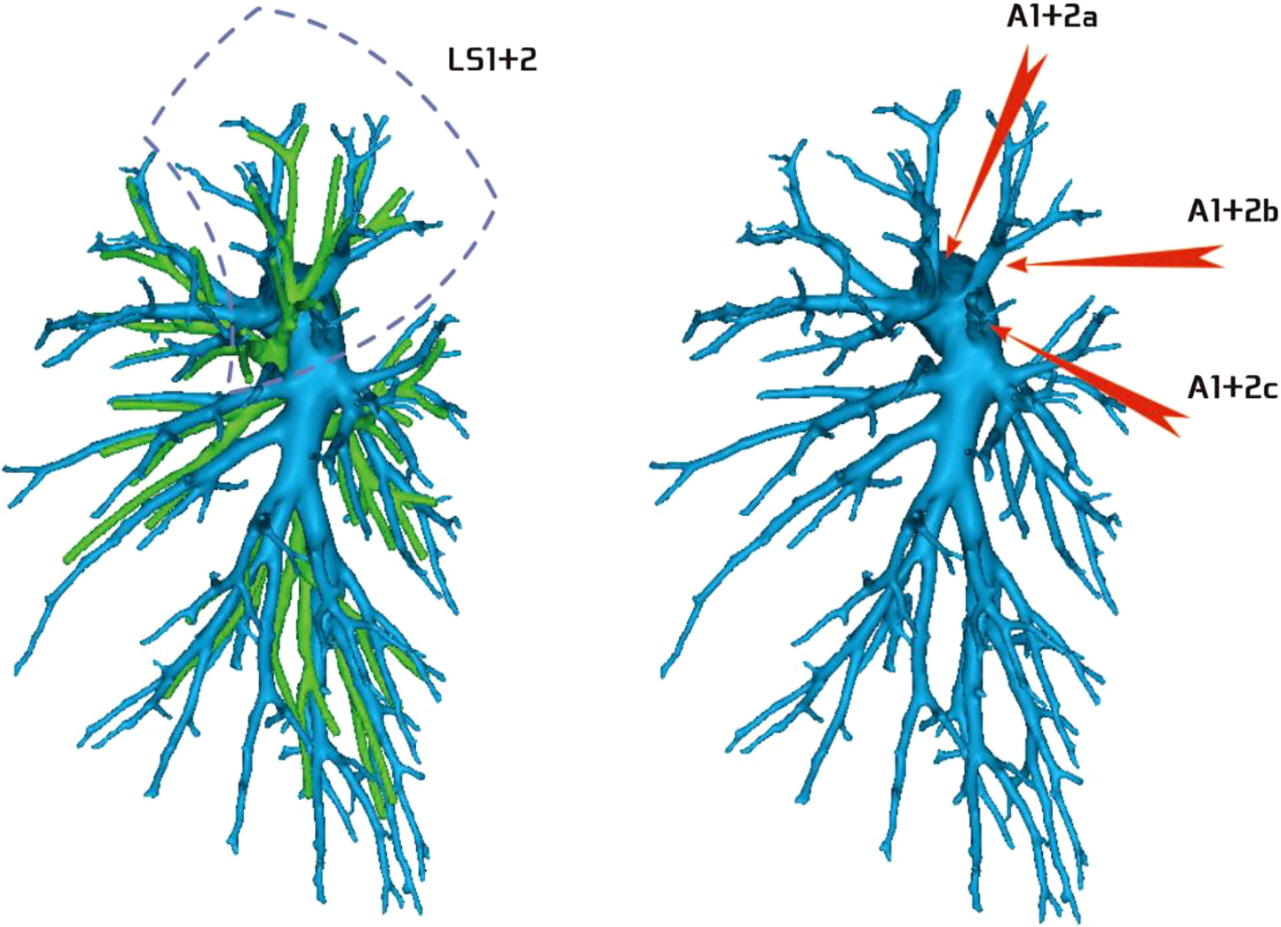
Type I: Dotted line is the LS1 + 2 area, and A(1 + 2)a, A(1 + 2)b, and A(1 + 2)c are separate branches.

**Figure 5:**
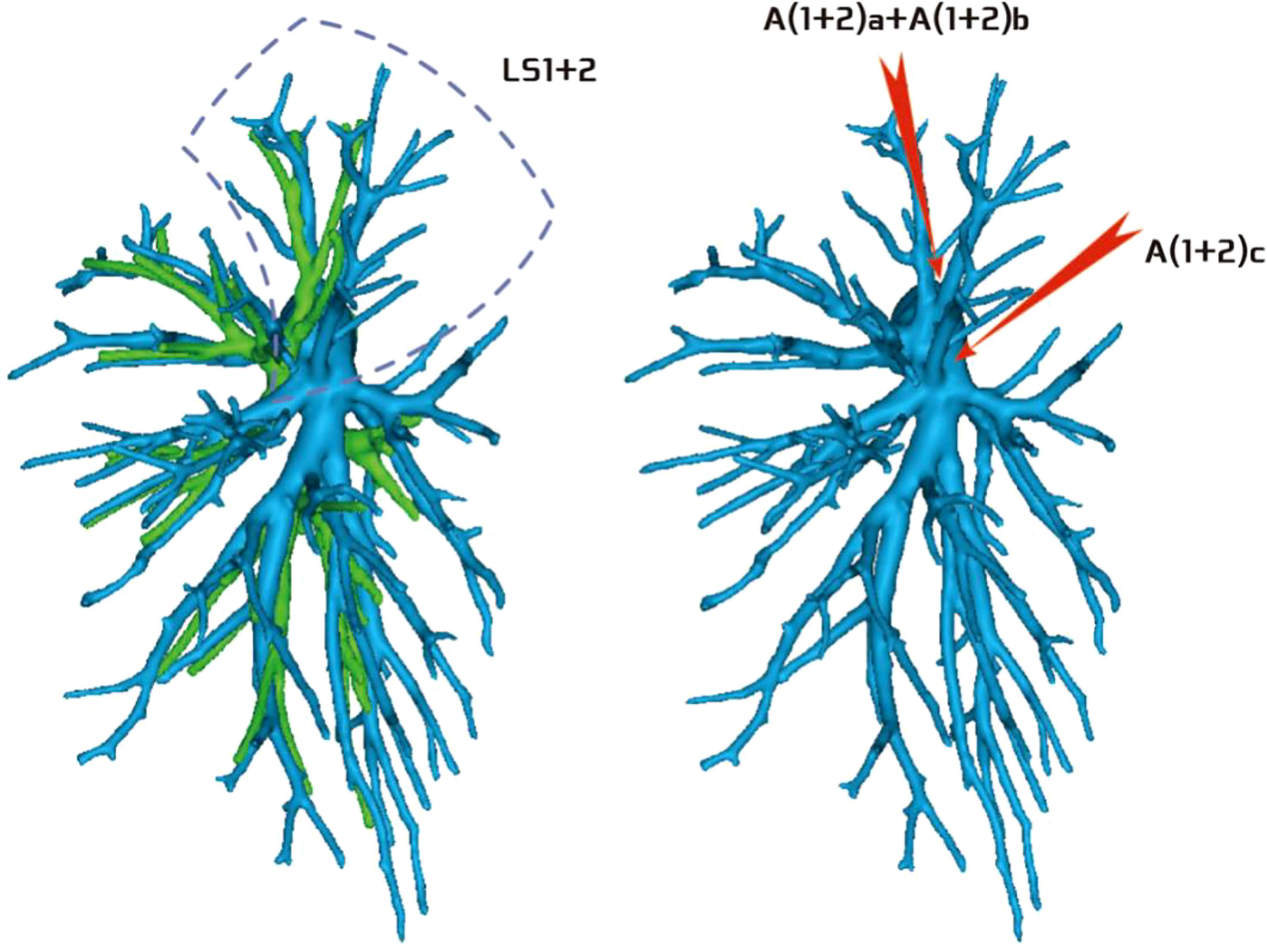
Type II: Dotted line is the LS1 + 2 area, A(1 + 2)a and A(1 + 2)b are co-trunks, and A(1 + 2)c is a single branch.

**Figure 6:**
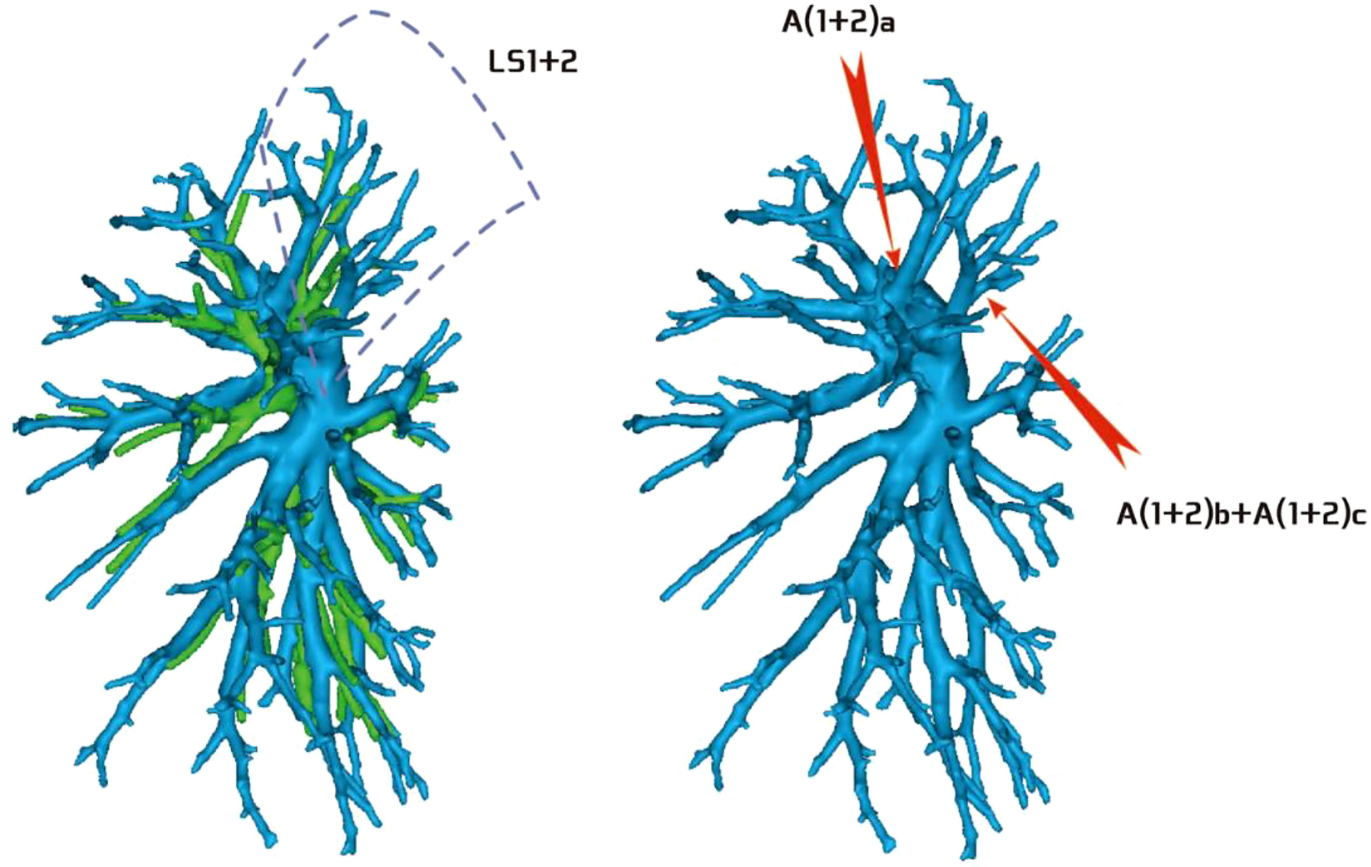
Type III: the dotted line is the LS1 + 2 area, A(1 + 2)a is a single branch, and A(1 + 2)b and A(1 + 2)c are co-trunks.

**Figure 7:**
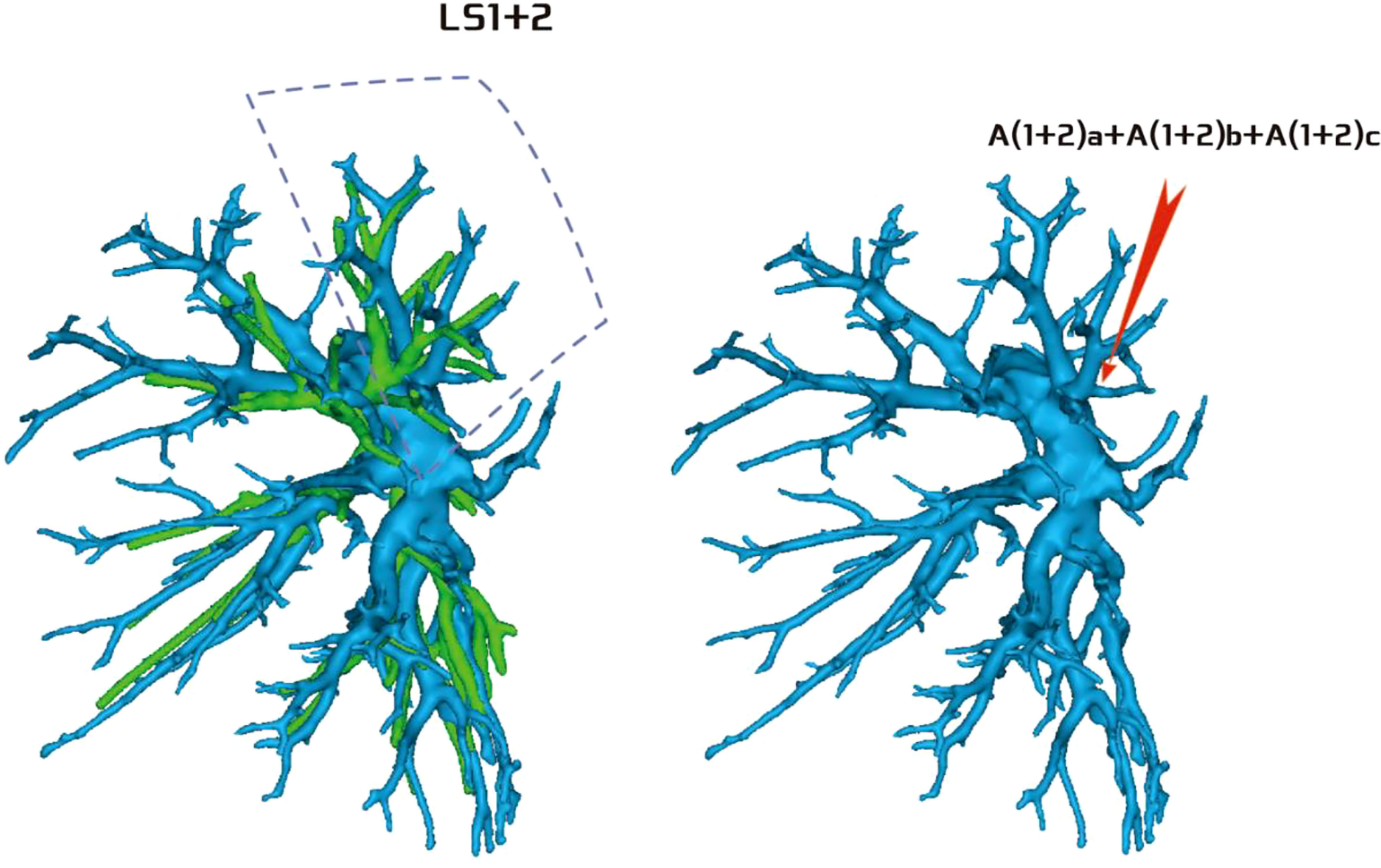
Type IV: Dotted line is the LS1 + 2 area, and A(1 + 2)a, A(1 + 2)b, and A(1 + 2)c are co-trunks.

Regarding the artery co-trunk of LS1 + 2, four subtypes were identified as follows: (1) in 35 patients, A(1 + 2)a, A(1 + 2)b, and A(1 + 2)c were separate branches (35/103, 33.9%) (**Figure 4**); (2) in 33 patients, A(1 + 2)a and A(1 + 2)b were co-trunks, and A(1 + 2) c was a single branch (33/103, 32.0%) (**Figure 5**); (3) in 21 patients, A(1 + 2)a was a single branch, and A(1 + 2)b and A(1 + 2)c were co-trunks (21/103, 20.3%) (**Figure 6**); (4) in 14 patients, A(1 + 2)a, A(1 + 2)b, and A(1 + 2)c were co-trunks (14/103, 13.5%) (**Figure 7**).

**Figure 8:**
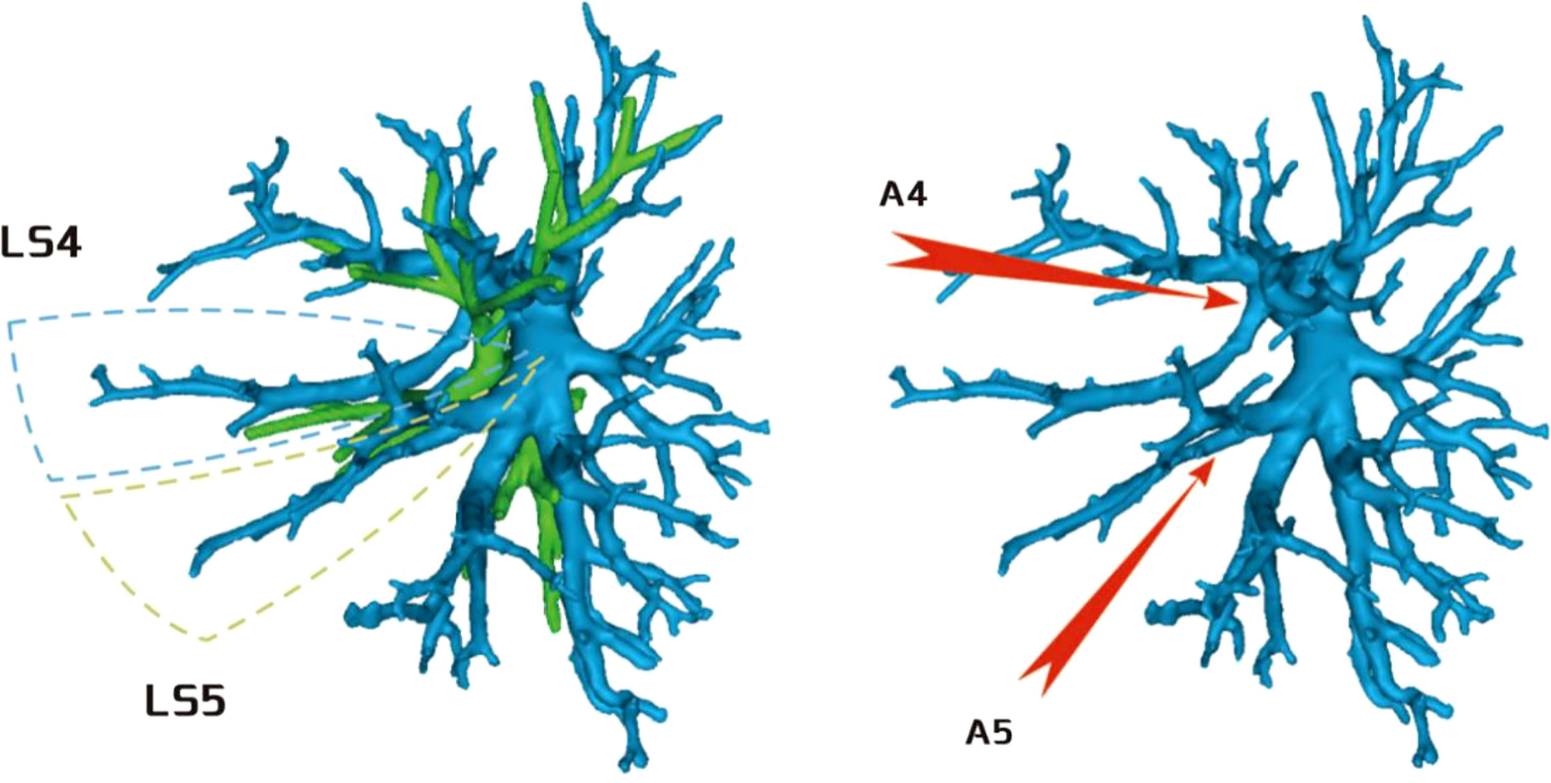
A4 originated from the mediastinal side of the lingular bronchus, and A5 originated from the oblique cleft side of the lingular bronchus.

**Figure 9:**
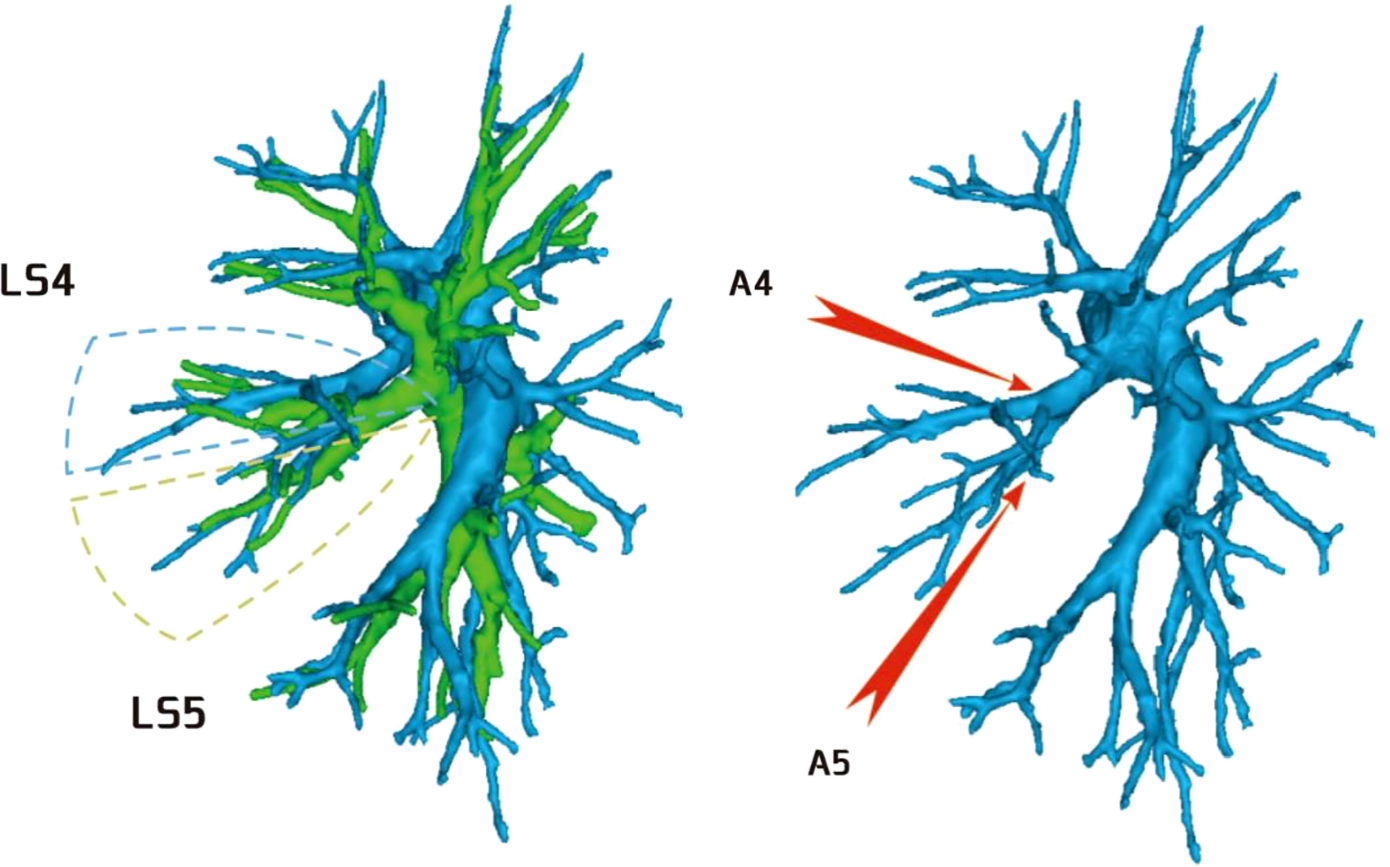
A4 + 5 originated from the mediastinal side of the lingular bronchus.

**Figure 10:**
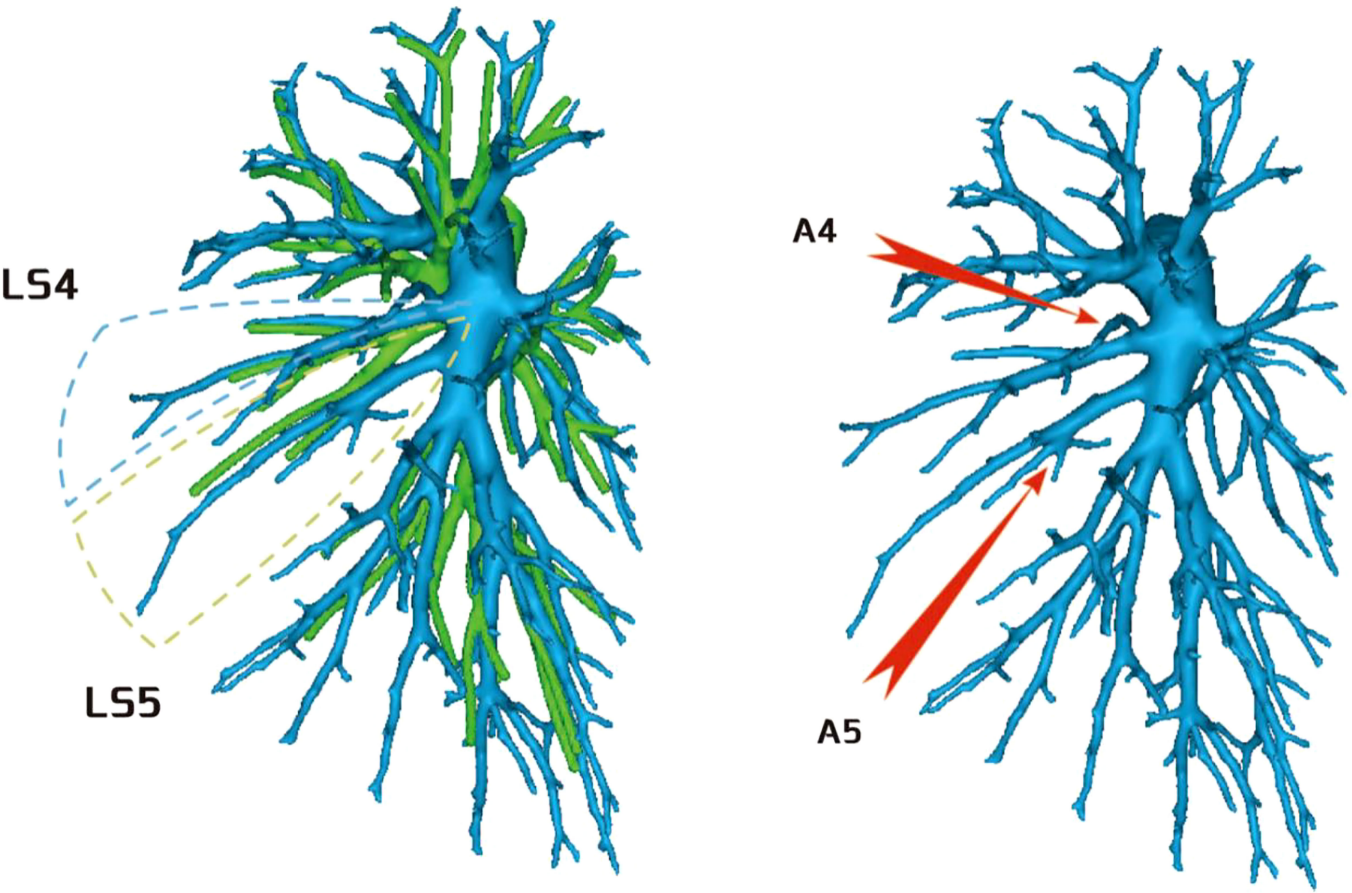
A4 + 5 originated from the oblique cleft side of the lingular bronchus.

Regarding the occurrence and walking of the lingular artery (A4 + A5), three subtypes were identified: (1) in 17 patients, A4 originated from the mediastinal side of the lingular bronchus, while A5 originated from the oblique cleft side of the lingular bronchus (17/103, 16.5%) (**Figure 8**); (2) in 7 patients, A4 + 5 originated from the mediastinal side of the lingular bronchus (7/103, 6.7%) (**Figure 9**); and (3) in all 79 patients, A4 + 5 originated from the oblique cleft side of the lingular bronchus (79/103, 76.7%) (**Figure 10**).

### Variations in the left upper pulmonary vein

The variations in the left upper pulmonary vein are listed in **Table 2**.

**Table 2:** Variations in the left upper pulmonary vein.

	Type	Subtype	Patients
Variations in the left upper pulmonary vein	The confluence and distribution of V(1 + 2)b + c	Descending central venous type: V(1 + 2)b + c trunk walked below B3	18
		No central venous type: V(1 + 2)b and V(1 + 2)c walked above and below B3, respectively	10
		Ascending central venous type: V(1 + 2)b+c trunk walked above B3	75
	The development of V(1 + 2)c*	Development of V(1 + 2)c* was relatively less	70
		Development of V(1 + 2)c* was relatively stout	33

V, Vein.

**Figure 11:**
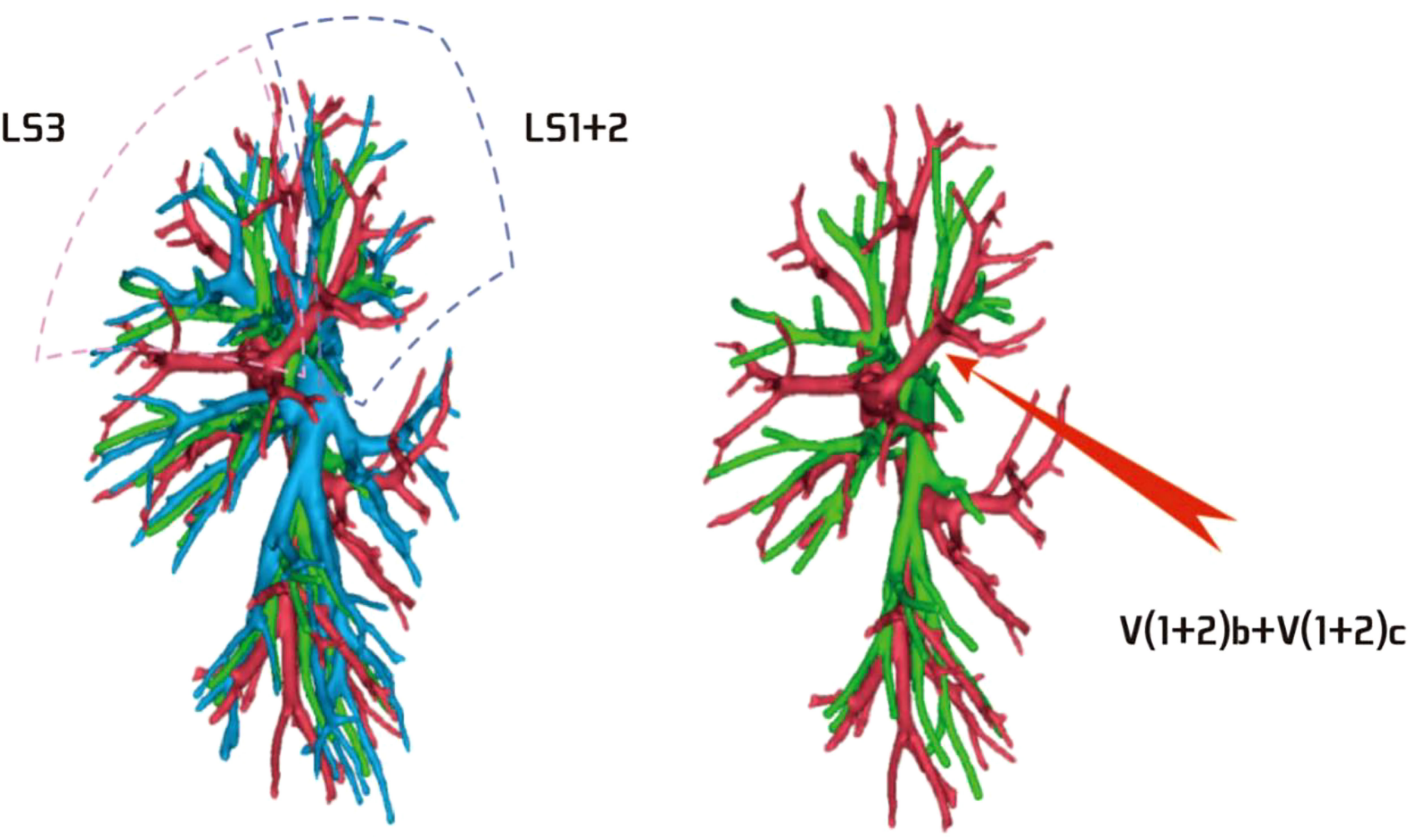
Descending central vein type: V(1 + 2)b + c backflow trunk walking below B3.

**Figure 12:**
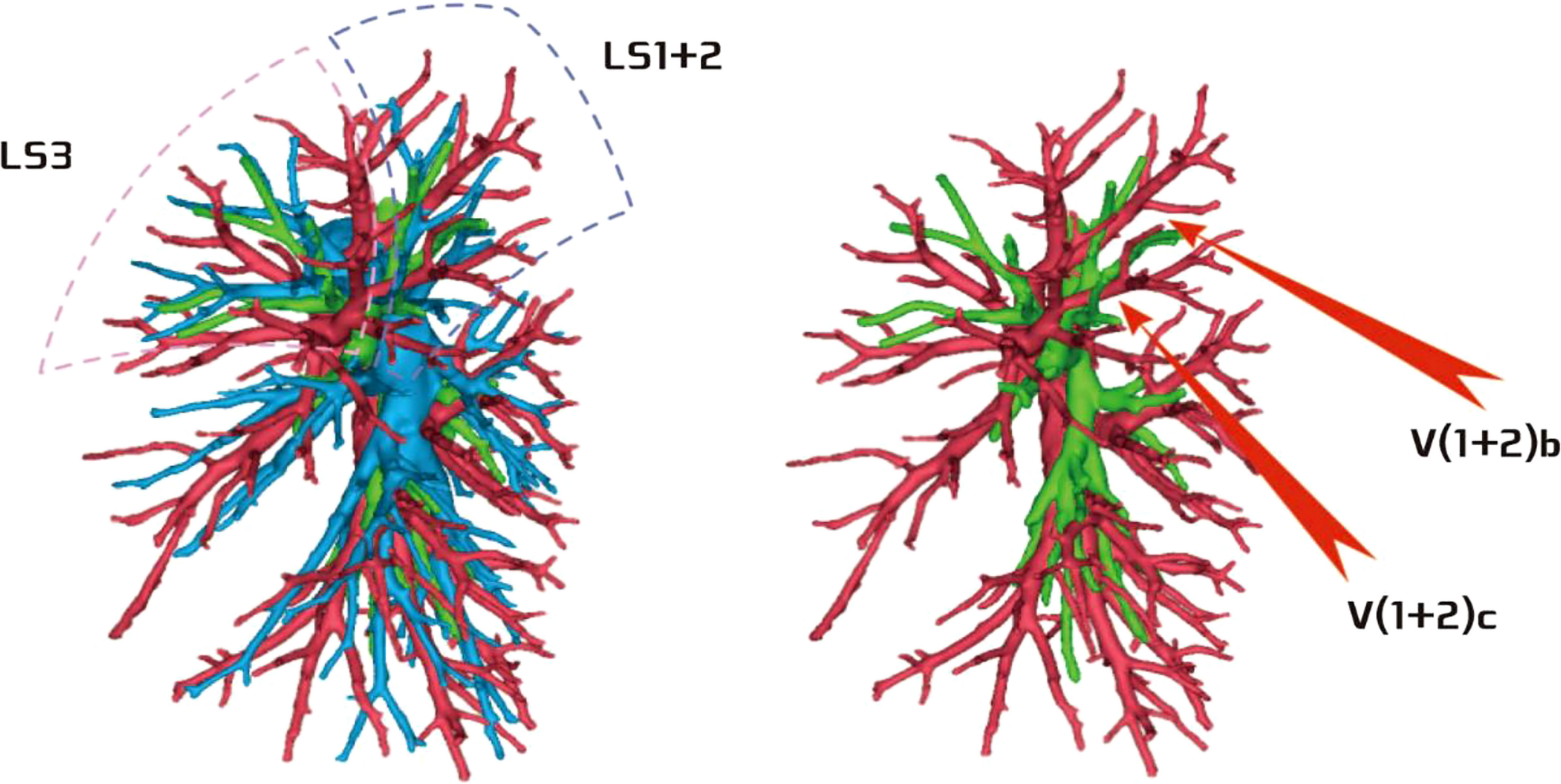
No central vein type: V(1 + 2)b and V(1 + 2)c walking above and below B3, respectively.

**Figure 13:**
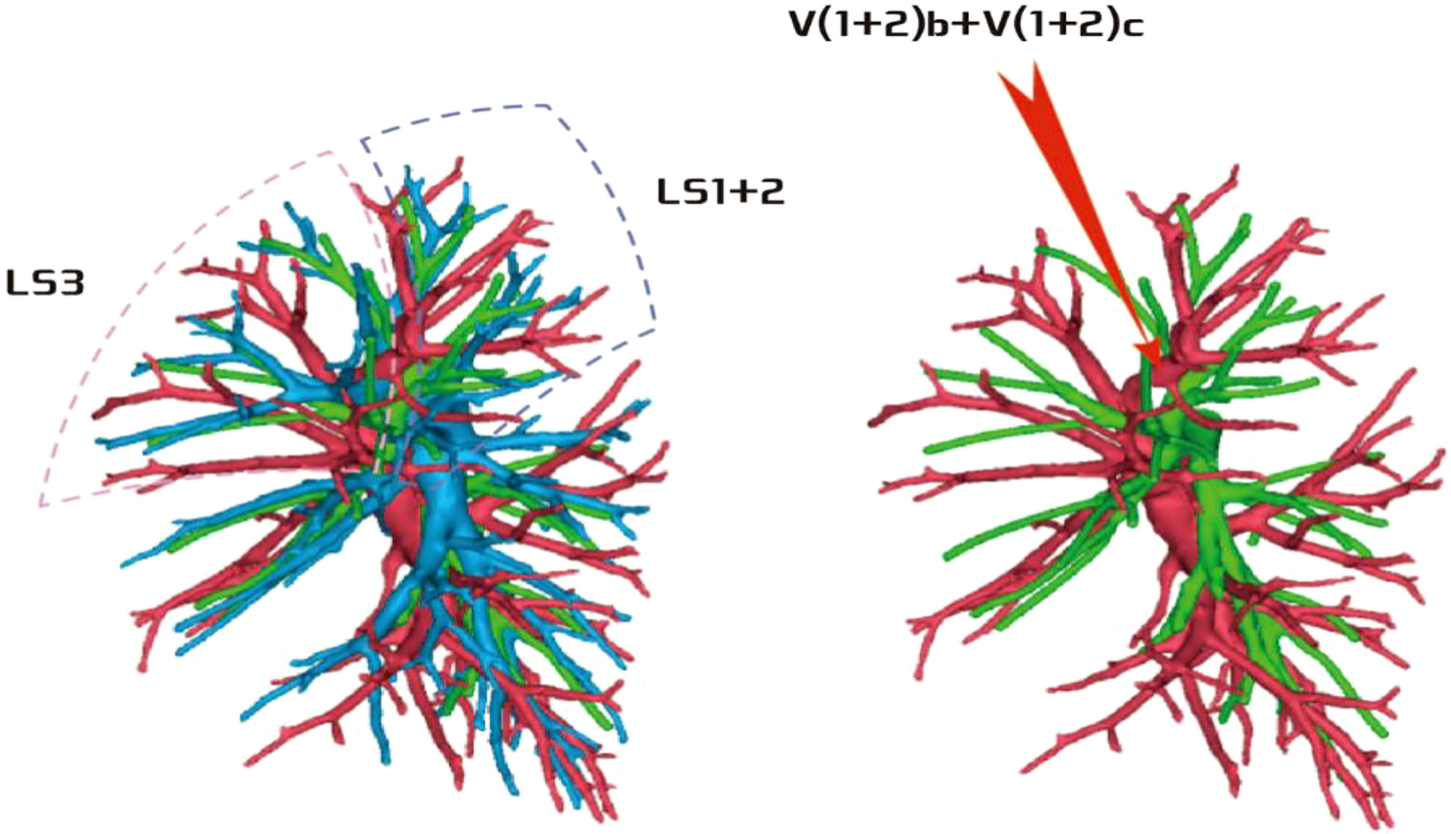
Ascending central venous type: V(1 + 2)b + c backflow trunk walking above B3.

The lingular segmental vein and V3 are relatively fixed and less variable and therefore were not described in detail. We took the existence of the central vein as the core and analyzed the situation of V1 + 2 according to its positional relationship with B3. The confluence and distribution of V(1 + 2)b + c had the following three situations: (1) 18 patients with descending central venous type: V(1 + 2) b + c backflow trunk walking below B3 (18/103, 17.4%) (**Figure 11**); (2) 10 patients with no central venous type: V(1 + 2)b and V(1 + 2)c walking above and below B3, respectively (10/103, 9.7%) (**Figure 12**); and (3) 75 patients had ascending central venous type: V(1 + 2)b + c reflux trunk walking above B3 (75/103, 72.8%) (**Figure 13**).

**Figure 14:**
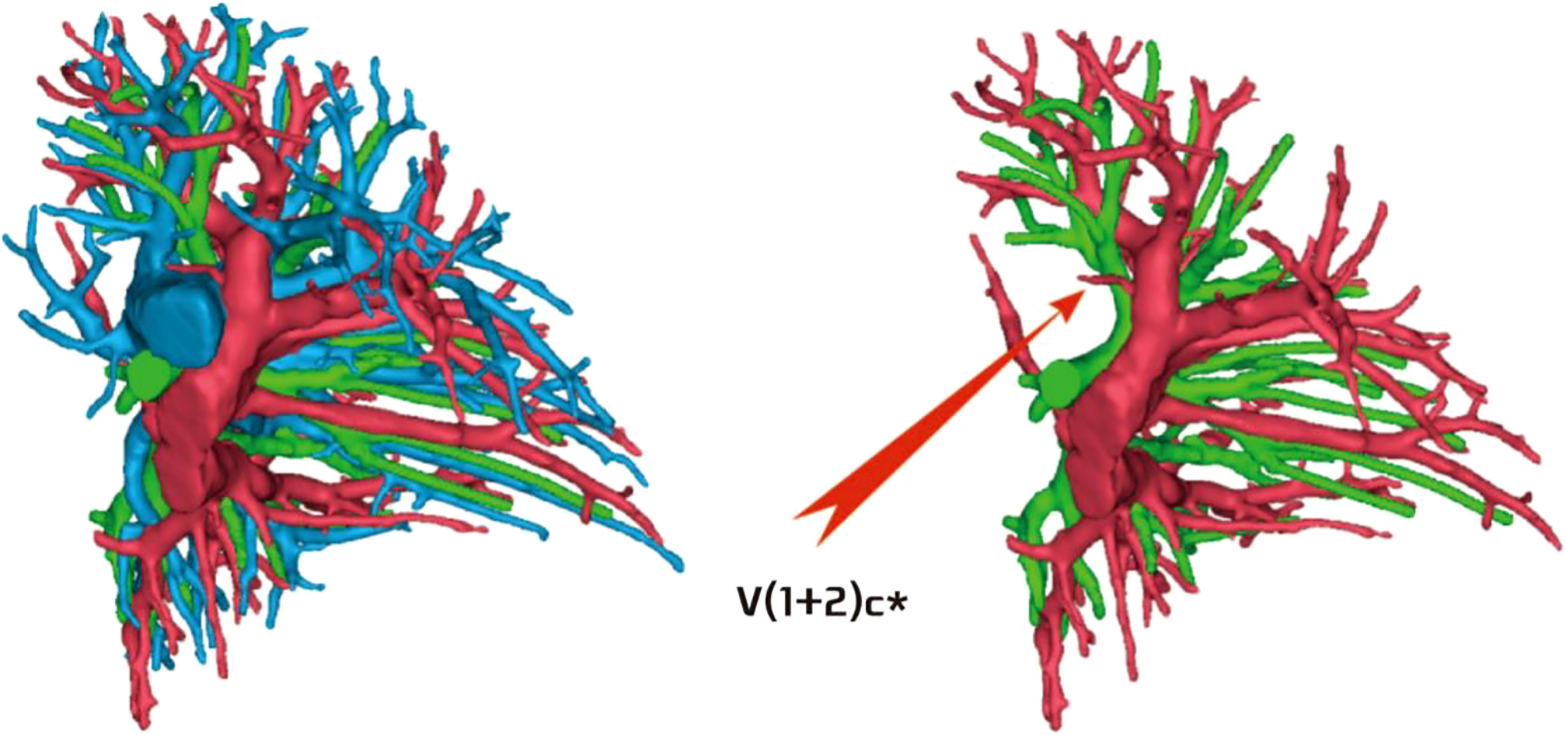
V(1 + 2)c* development was relatively less [V(1 + 2)c* vessel diameter greater than 1 mm and length less than or equal to 30 mm].

**Figure 15:**
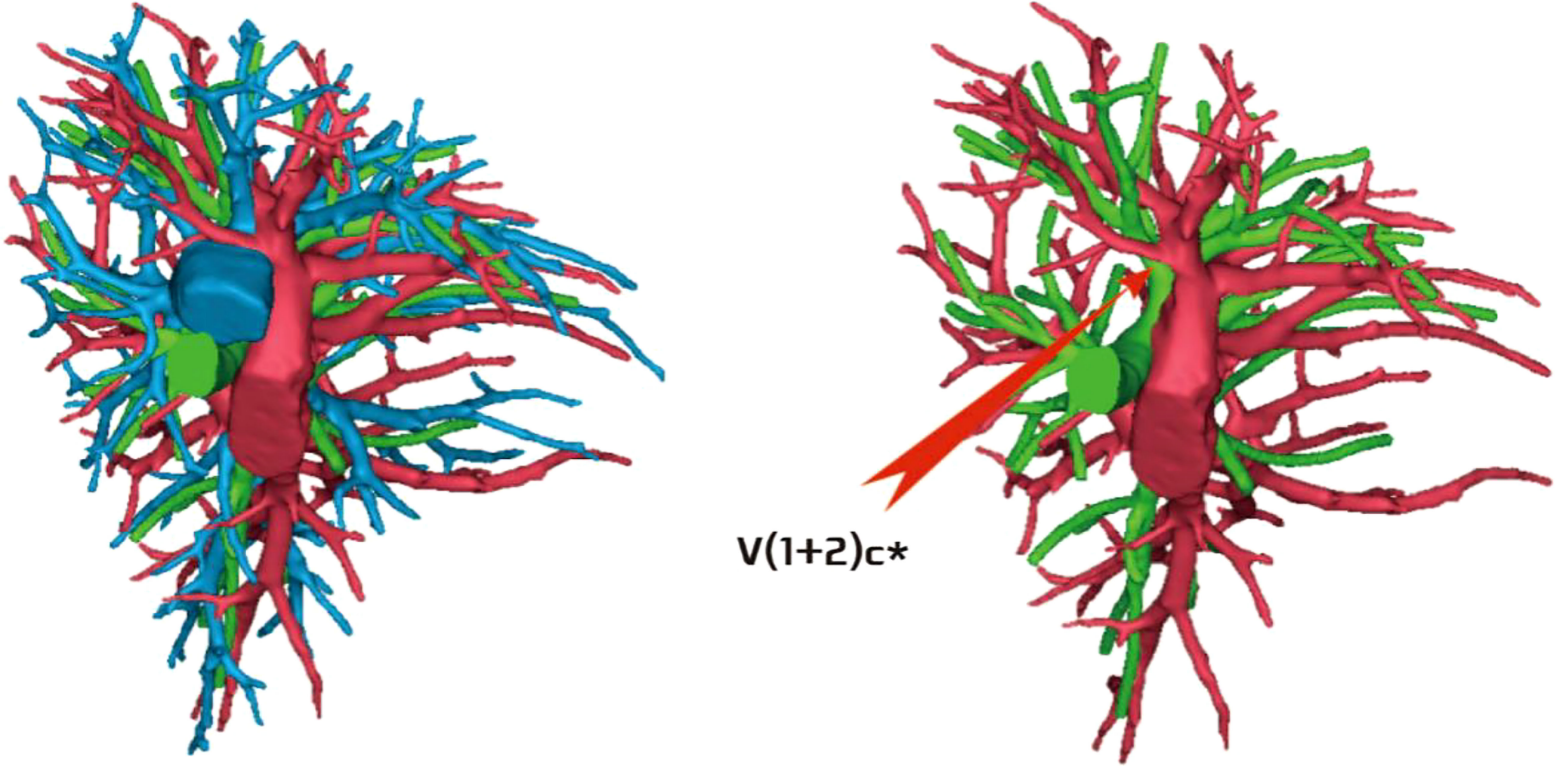
V(1 + 2)c* was relatively stout [V(1 + 2)c* vessel diameter was greater than 1 mm and length was greater than 30 mm].

The development of V(1 + 2)c* had the following two situations: (1) The development of V(1 + 2)c* in 70 patients was relatively less. We defined it as the diameter of V(1 + 2)c* blood vessel larger than 1 mm and the length less than or equal to 30 mm (70/103, 68.0%) (**Figure 14**); and (2) in 33 patients, V(1 + 2)c* development was relatively stout. We defined the development of stout as V(1 + 2)c* vessel with a diameter greater than 1 mm and a length greater than 30 mm (33/103, 32.0%) (**Figure 15**).

### Variations in the left upper lobe bronchus

The variations in the left upper lobe bronchus are listed in **Table 3**.

**Table 3:** Variations in the left upper lobe bronchus.

	Type	Subtype	Patients
Variation of the left upper lobe bronchus	Bifurcation type	Bifurcated type A	62
		Bifurcated type B	2
		Bifurcated type C	4
		Bifurcated type D	2
		Bifurcated type E	6
		Bifurcated type F	2
		Bifurcated type G	14
	Trifurcation type	Trifurcated type H	11

**Figure 16:**
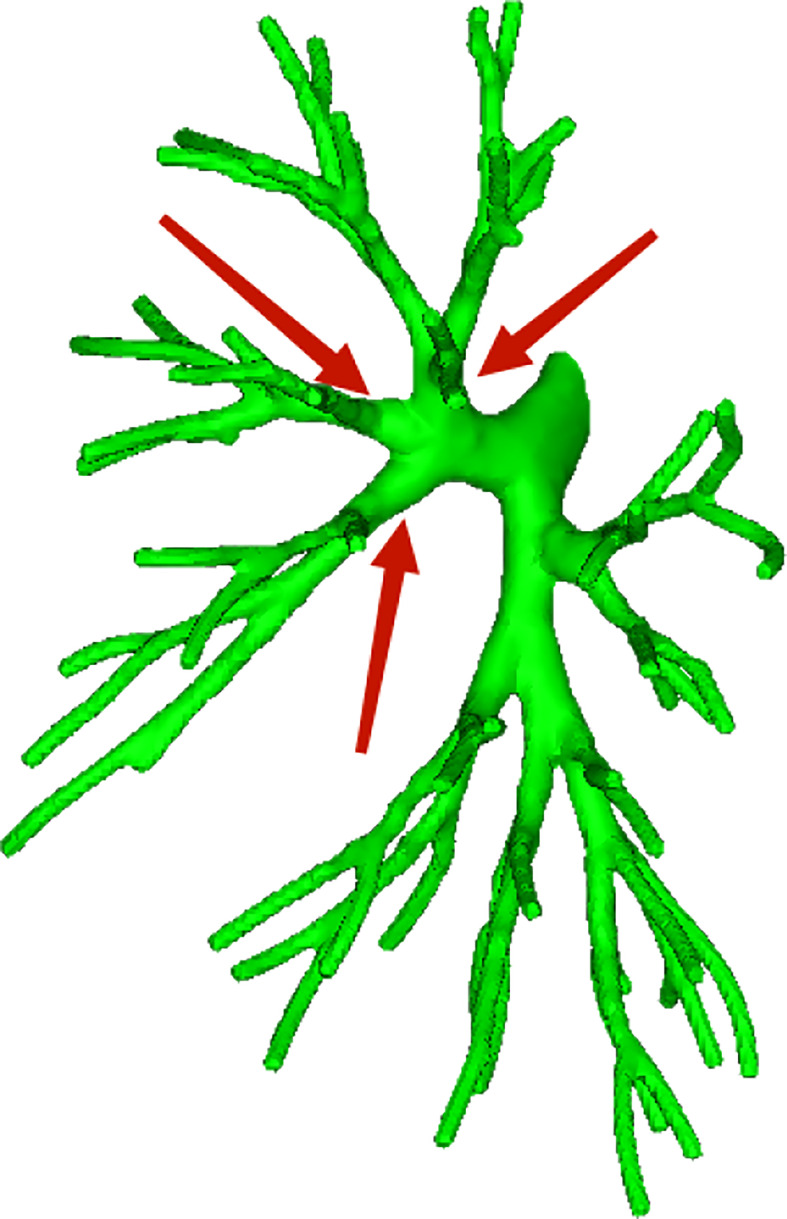
Trifurcation type; the branch mode was B1 + 2, B3, and B4 + 5.

**Figure 17:**
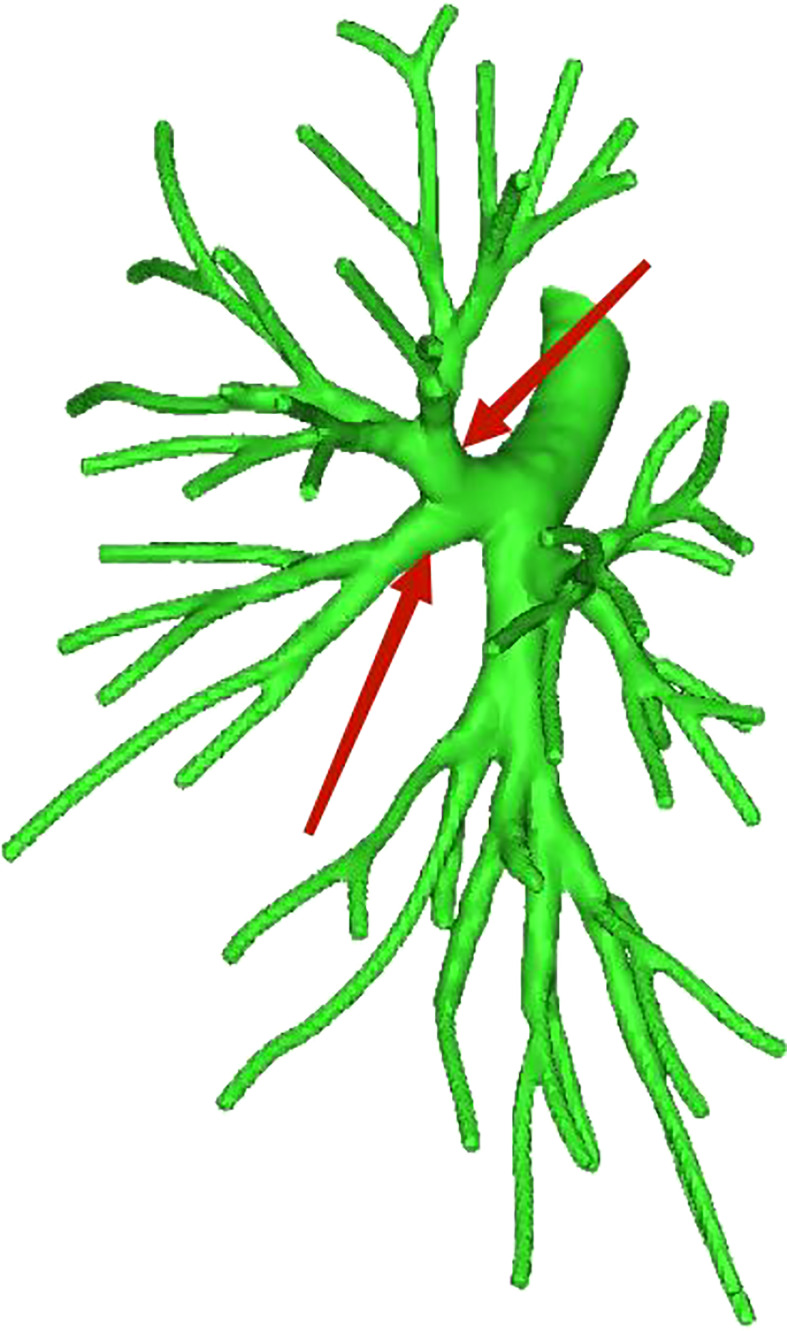
Bifurcation type; the branch mode was B1 + 2 + 3 and B4 + 5.

If the upper lobe bronchus of the left lung was analyzed from the secondary bronchial trunk, it was preliminarily divided into two types: (1) 11 patients had trifurcation type, and the branching pattern was B1 + 2, B3, and B4 + 5 (11/103, 10.7%) (**Figure 16**); and (2) 92 patients had bifurcation type, and the branching pattern was B1 + 2 + 3 and B4 + 5 (92/103, 89.3%) (**Figure 17**).

**Figure 18:**
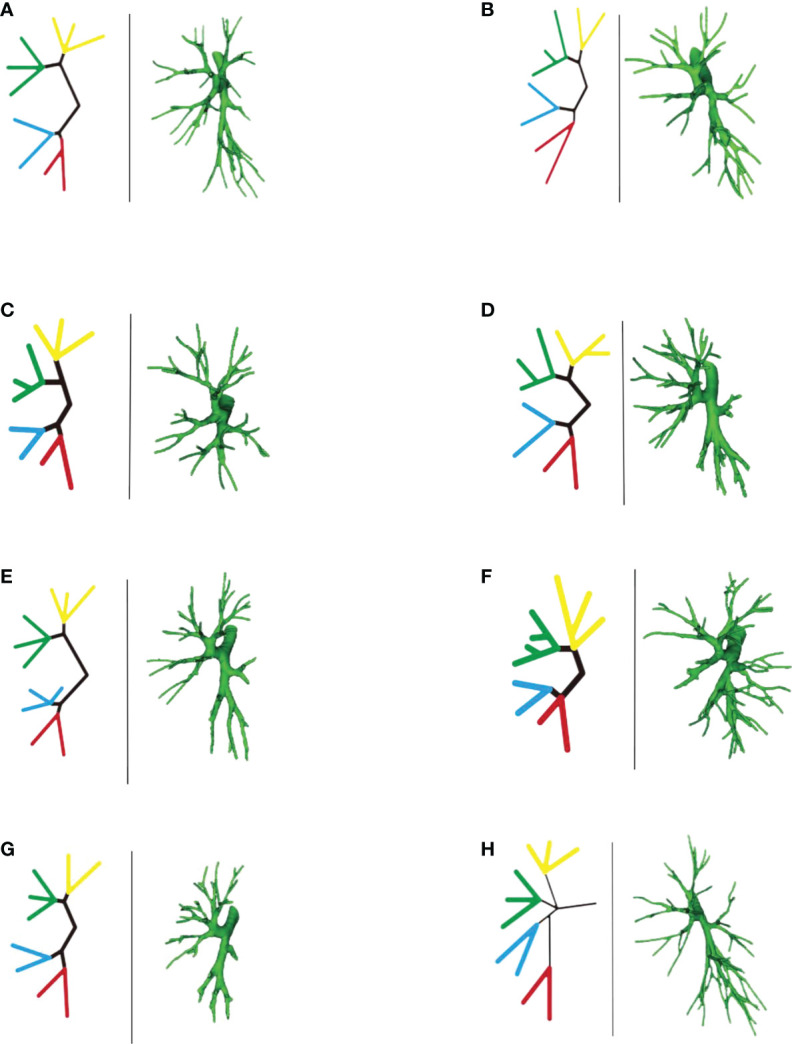
**(A-H)** Left part is a simplified schematic diagram of the left upper lobe bronchus, and the right part is the specific reconstruction of the left whole lung bronchus.

According to the subsegmental bronchial co-trunk distribution, it was further subdivided into the following eight types:

Bifurcation type A (**Figure 18A**) (62/103, 60.2%): The B1 + 2 + 3 branch was divided into B1 + 2 branch [B1 + 2 bronchus divided into B(1 + 2)a, B(1 + 2)b, and B(1 + 2)c single branches] and B3 branch (B3 bronchus divided into B3a, B3b, and B3c single branches). The B4 + 5 branch was divided into B4 and B5 branches, the B4 branch was divided into B4a and B4b branches, and the B5 branch was divided into B5a and B5b branches.

Bifurcation type B (**Figure 18B**) (2/103, 1.9%): B1 + 2 + 3 was first divided into two branches: B1 + 2 and B3. Among these, B1 + 2 was subdivided into two sub-branches, but it was difficult to distinguish the B(1 + 2)b branch because part of the bronchus of B(1 + 2)b and B(1 + 2)a co-trunk and the other part of the bronchus was connected with B(1 + 2)c. The B3 branch was divided into two branches, B3a and B3b were co-trunks, and B3c was a separate branch. The B4 + 5 branch was divided into B4 and B5 single branches. Then, the B4 branch was divided into B4a and B4b branches, and the B5 branch was divided into B5a and B5b branches.

Bifurcation type C (**Figure 18C**) (4/103, 3.8%): The B1 + 2 + 3 branch was divided into one branch of B1 + 2 and one branch of B3. B1 + 2 branches were B(1 + 2)a, B(1 + 2)b, and B(1 + 2)c each. B3 branch was B3c 1 branch, and B3a + b were co-trunks into one branch.

Bifurcation type D type (**Figure 18D**) (2/103, 1.9%): The B1 + 2 + 3 branch was divided into two branches (B1 + 2 and B3). Then, the B1 + 2 branch was divided into B(1 + 2)a and B(1 + 2) b + c branches. The B3 branch was divided into B3c and B3a + b branches. The B4 + 5 branch was divided into B4 and B5 branches, the B4 branch was divided into B4a and B4b branches, and the B5 branch was divided into B5a and B5b branches.

Bifurcation type E (**Figure 18E**) (6/103, 5.8%): B1 + 2 + 3 was first divided into B1 + 2 and B3 branches. Then, the B1 + 2 branch was divided into B(1 + 2)a, B(1 + 2)b, and B(1 + 2)c branches. The B3 branch was divided into B3a, B3b, and B3c branches. However, B4 had variations. The B4 + 5 branch was divided into B4 and B5 branches, and the B4 branch was divided into B4a, B4b, and B4* branches. The B5 branch was divided into two B5a and B5b branches.

Bifurcation type F (**Figure 18F**) (2/103, 1.9%): The B1 + 2 + 3 branch was divided into B1 + 2 and B3 branches. The B1 + 2 branch was divided into B(1 + 2)a, B(1 + 2)b, and B(1 + 2)c branches. However, the B3 branch was more special (antler four branch types). The B3 branch was composed of B3c + B3bi and B3a + B3bii. The B4 + 5 branch was divided into B4 and B5 branches. The B4 branch was divided into B4a and B4b branches, and the B5 branch was divided into B5a and B5b branches.

Bifurcation type G (**Figure 18G**) (14/103, 13.6%): It was similar to B type, but the B3 branch was different: B1 + 2 + 3 was first divided into B1 + 2 and B3 branches. Among these, the B1 + 2 branch was divided into two branches, and it was difficult to distinguish the B(1 + 2)b branch because part of the bronchi of B(1 + 2)b and B(1 + 2)a were co-trunks, and the other part of the bronchus was connected with B(1 + 2)c. However, the B3 branch was divided into B3a, B3b, and B3c branches. The B4 + 5 branch was divided into B4 and B5 branches. The B4 branch was divided into B4a and B4b branches, and the B5 branch was divided into B5a and B5b branches.

Trifurcation type H (**Figure 18H**) (11/103, 10.6%): The upper lobe bronchus developed into three branches directly: B1 + 2 were co-trunks into 1 branch, 1 branch was B3, and B4 + 5 were co-trunks into 1 branch.

## Discussion

Low-dose thin-slice chest CT has greatly improved early-stage lung cancer screening rates. With the advancement of the Japan Clinical Oncology Group (JCOG) 0802 and 0804 studies (3, 4), an increasing number of scholars have recognized the advantages of anatomical segmentectomy in protecting lung function. At the same time, anatomical segmentectomy has become an important treatment for early-stage NSCLC. However, anatomical segmentectomy is not a simple technique. It requires the surgeon to have a full understanding of the anatomy of the lung segment and requires skills during the surgery. Therefore, it is extremely necessary to read the chest CT in detail before the surgery, especially the three-dimensional reconstruction of the lung based on CT images (5). The left upper lobe is the largest lung lobe in the human body, and it also has the most anatomical variation (6). Reports of variations similar to those in the mediastinal lingular segmental artery are available (7), but a few systematic reports on the variations in the left upper lobe blood vessels and bronchus also exist. The anatomy of the upper lobe of the left lung is complex, and it is currently customary to divide the upper lobe of the left lung into the proper segment [including apical posterior segment (LS1 + 2) + anterior segment (LS3)] and the lingular segment [including upper lingular segment (LS4) + lower lingular segment (LS5)]. In clinical practice, many scholars tend to equate the proper segment with the upper lobe of the right lung and the lingular segment with the middle lobe of the right lung to facilitate anatomical resection (8). The core of segmentectomy is the identification of the lung segment where the lesion is located, and the key to the surgery is the treatment of arteries and bronchi and intersegmental veins. This study reviewed the 3D reconstruction data of 103 patients who underwent anatomical left upper lobe segmentectomy in our center to better carry out anatomical left upper lobe segmentectomy. The left upper lobe was first divided into the proper segment (LS1 + 2 and LS3) and the lingular segment (LS4 and LS5) to facilitate understanding, and a preliminary experience summary was made.

Pulmonary artery management is often the first step in segmentectomy. In this study, the branches of the left upper pulmonary lingular artery branch into the LS1+2 and LS3 segments had the following three variations (Figs 1–3), and the frequency of occurrence was not high (21/103, 20.3%). The analysis of the arterial system showed that the arterial system of the proper segment and the lingular segment was relatively fixed, with a few lingular segment arterial branches into the proper segment lung tissue. Therefore, from the perspective of the arteries, taking the proper segment and the lingular segment as a whole was reasonable.

In this study, A1 + 2 branch had four types of branches (Figs 4–7). The most common was type I (**Figure 4**). For such cases, when the lesion was relatively limited and only located in one of the sub-segments of the LS1 + 2, such as the LS(1 + 2)a sub-segment, dealing with its arteries might be safer and more convenient because dissecting the branches more distally was not required. For the case of type II (**Figure 5**) or type III (**Figure 6**), we could understand that the LS(1 + 2) a + b or the LS(1 + 2) b + c was the dominant segment. When the lesion was in the dominant segment we could perform subsegmental resection as a whole, which was more convenient and safer. In the case of type IV (**Figure 7**), it was more convenient to perform the LS1 + 2 segmentectomy. However, when a subsegment needed to be removed, it was necessary to further dissociate the branch artery at the distal end, which greatly increased the difficulty in dissection.

Regarding the anatomy of the lingular artery, the most common distribution of the lingular artery in this study was that the lingular artery originated from the oblique cleft side of the lingular bronchus (**Figure 10**). When performing lingular segmentectomy, when the oblique fissure was well developed, it was convenient and safe to process the lingular artery from the side of the oblique fissure, followed by the lingular bronchus. However, when the oblique fissure was poorly developed, it was often necessary to first free the lingular bronchus from the side of the hilum of the mediastinum. However, it was essential to avoid causing damage to the lingular artery when the bronchus was freed. At the same time, it might be interfered with by the superior lobe venous system, and the lingular vein might be treated first to prevent bleeding.

For the case where A4 + 5 originated from the mediastinal side of the lingular bronchus (**Figure 9**), when the oblique fissure was well developed, it was easier to treat the lingular bronchus from the pulmonary fissure first. However, it was still necessary to protect the lingular artery on the mediastinal side. When the oblique fissure was poorly developed, it might be more reasonable to first treat the lingular artery from the anterior mediastinal hilum. However, the treatment was often interfered with by the venous system, and the lingular vein might need to be treated first.

For the case where A4 originated from the mediastinal side of the lingular bronchus and A5 originated from the oblique fissure side of the lingular bronchus (**Figure 8**), the processing was more complicated, and it might be necessary to deal with the anterior hilum and the oblique fissure, respectively, while dissecting A4 and A5. Or we need to start from the hilum or oblique fissure and proceed in a single direction (artery → bronchi → artery), but consider the interference of the upper lobe venous system; starting from the mediastinal side first may be difficult. Therefore, it is still recommended to treat the arteries and bronchus first from the oblique fissure side when the fissure is well developed.

In addition, some scholars tried to analyze the variations in the mediastinal lingular artery. The lingular segment was sent out before the proper segment artery, and the incidence rate was reported as about 5.6% (9). Such cases often required free arteries from the anterior mediastinal hilar side. The core anatomical link considering segmentectomy was still the bronchus. Therefore, the analysis was carried out through the relationship between the lingular bronchus and the lingular artery in this study.

The pulmonary venous return system is an important part of segment function. The anatomy of V3 and V4–5 is relatively fixed and has fewer variations and The variation of V1 + 2 is more complicated (8). According to the reconstruction, we used the central vein of the upper lobe as the anatomical core and analyzed the veins in the posterior apical segment according to its positional relationship with the anterior segment bronchus (B3). For the confluent distribution of V(1 + 2)b + c, three types were identified: (1) descending central venous type (**Figure 11**); (2) no central venous type (**Figure 12**); and (3) ascending central venous type (**Figure 13**). For V(1 + 2)c*, most cases were small developments (70/103, 68.0%) (**Figure 14**). It was seen that the venous return of the proper segment mainly depends on V(1 + 2)b+c in the venous system of the upper lobe.

For the noncentral venous type (**Figure 12**), the patients needed to be accurately identified during segmentectomy because the wrong amputation might be caused by the absence of dominant veins (especially the noncentral venous type, with or without the small development of V1 + 2c*), and it affected the residual lung function by assisting in the reflux. In the case of central veins, it was recommended to retain as much as possible in the subsegmental resection of the proper segment, as long as the resection margin was sufficient.

In segmentectomy, the previous recommendation was to preserve as many veins as possible to reduce reflux obstruction to the residual lung (2). At the same time, clinically, whether relying on indocyanine green fluorescence microscopy to display the lung segment plane or on the lung ventilation collapse method (after cutting the artery or after the artery and the bronchus are severed at the same time, performing lung ventilation collapse), it is enough to show appropriate segmental planes (10, 11). Therefore, combined with the aforementioned analysis of the venous situation, the anatomical significance of the pulmonary veins is more of a protective guide for surgeries, especially the central venous type, as long as they comply with the principles of oncology (such as sufficient incision margins).

Finally, the core link between lung segment confirmation and segmentectomy is the identification of the bronchus. Based on the condition of the anterior arteries and veins, we continued to analyze the bronchus by taking the proper segment and the lingular segment as a whole. According to the distribution of secondary bronchi, the common trunk types of the left upper lobe bronchus were initially divided into two types: (1) trifurcation type (**Figure 16**); and (2) bifurcation type (**Figure 17**).

The distribution showed that most of the bronchial types of the left upper lobe were bifurcated types (**Figure 17**), further indicating that, from the perspective of the bronchus, scholars artificially took the proper segment as a whole and compared it with the right upper lobe; the tongue segment as a whole was more feasible compared with the right middle lobe. When a large lesion existed and it was difficult to ensure the margin of subsegmental resection, proper segmentectomy or lingular segmentectomy was also more in line with the principles of oncology and could relatively preserve healthy lung tissue.

We further subdivided the bronchus into eight types according to the subsegmental bronchial co-trunk to better understand the anatomical variations in bronchi (**Figure 18A-H**). The aforementioned data showed that, according to the subsegmental bronchial declassification, seven cases had bifurcated type (**Figure 18**A/B/C/D/E/F/G). In contrast, trifurcation type H (**Figure 18H**) occurred at a low frequency, and relative variations were relatively rare. The subdivision of the variation in the trifurcation type was difficult in this study due to the limited sample size.

Among the bifurcated types, the most common was bifurcated type A (**Figure 18A**), which we called the “classic type” in this study. It was also the clearest type in clinical anatomy: The B1 + 2 + 3 branch was divided into B1 + 2 [B(1 + 2)a, B(1 + 2)b, and B(1 + 2)c] and B3 (B3a, B3b, and B3c) branches. The B4 + 5 branch was divided into B4 and B5 branches, the B4 branch was divided into B4a and B4b branches, and B5 the branch was divided into B5a and B5b branches. In this type, whether in segmentectomy or subsegmentectomy, the anatomical identification was relatively easy, and it was better to determine the extent of resection.

The bifurcated B type (**Figure 18B**) and the bifurcated G type (**Figure 18G**) were generally similar. Both belonged to the type with unclear development of the B(1 + 2) b branch, but the B3 branch was different. In such cases, the subsegmental resection of LS1 + 2, especially LS(1 + 2)b subsegmental resection, was difficult.

Regarding the situation of the lingular bronchus, the branch variation was relatively rare. In the A/B/C/D/F/G classification, the lingular bronchial branches were all B4 + 5 branches. The B4 + 5 divided into B4 and B5 branch, then B4→B4a, B4b branch, B5→B5a, B5b branch. Only the bifurcated E type (**Figure 18E**) was special. The B4 branch was divided into three branches and belonged to the lingular bronchus variant type, but the frequency of occurrence was not high. In general, the identification and management of the bronchial tubes were relatively easy during lingular segmentectomy or sub-segmentectomy.

The bifurcated F type (**Figure 18F**) was a special variant of the B3 branch (according to the shape, we called it antler 4 branch type): This type was characterized by the B3 branch as B3c + B3bi and B3a + B3bii. It was difficult to perform LS3b subsegment resection alone because the branches of B3b were formed from the branches of B3a and B3c.

Finally, a clinical statement shows that the pulmonary artery, in segmental level, is always accompanied by the bronchus, which is more stable in other lobes, especially the right upper lobe (12). Interestingly, in the upper left lobe, especially in the subsegmental anatomy, this accompaniment does not seem to be strict. For example, in this study, the most common type of bronchus was bifurcated type A (**Figure 18A**) (62/103, 60.2%); the B1 + 2 + 3 branch was divided into B1 + 2 [B(1 + 2) a, B(1 + 2)b, and B(1 + 2)c branches. Correspondingly, A(1 + 2)a, A(1 + 2)b, and A(1 + 2) c were separate branches (35/103, 33.9%, **Figure 4**), but the distribution ratios of the two were quite different (60.2% vs 33.9%). It is expected that larger data will be verified in the future.

In summary, preoperative CT-based three-dimensional reconstruction of pulmonary arteries, veins, and bronchi is of great significance. It can help understand the variations, accurately locate lesions before surgery, and effectively plan operations (such as the processing sequence of blood vessels and bronchial tubes, resection of blood vessels, and resection range determination), thus assisting the beginners to strengthen their understanding of the anatomy of the upper left lobe. The limitation of this study was that the number of samples was small, which is expected to be improved in future work.

## Data availability statement

The raw data supporting the conclusions of this article will be made available by the authors, without undue reservation.

## Author contributions

YD: project design and initiation, data analysis, manuscript writing CH: data collection SC: data collection WL: data collection LD: data collection RJ: data collection CW: data collection SL: data collection XY: data collection XYY: data collection YY: data collection CY: data collection HZ: data collection ZW: data collection LW: data collection KM: data collection ZY: supervisor XG: supervisor. All authors contributed to the article and approved the submitted version.

## Funding

Supported by Shenzhen High-level Hospital Construction Fund, Shenzhen Key Medical Discipline Construction Fund(No. SZXK075) and Sanming Project of Medicine in Shenzhen(No. SZSM201612097).

## Conflict of interest

The authors declare that the research was conducted in the absence of any commercial or financial relationships that could be construed as a potential conflict of interest.

## Publisher’s note

All claims expressed in this article are solely those of the authors and do not necessarily represent those of their affiliated organizations, or those of the publisher, the editors and the reviewers. Any product that may be evaluated in this article, or claim that may be made by its manufacturer, is not guaranteed or endorsed by the publisher.
